# Left ventricular diastolic function associated with common genetic variation in *ATP12A* in a general population

**DOI:** 10.1186/s12881-014-0121-6

**Published:** 2014-11-04

**Authors:** Judita Knez, Erika Salvi, Valérie Tikhonoff, Katarzyna Stolarz-Skrzypek, Andrew Ryabikov, Lutgarde Thijs, Daniele Braga, Malgorzata Kloch-Badelek, Sofia Malyutina, Edoardo Casiglia, Danuta Czarnecka, Kalina Kawecka-Jaszcz, Daniele Cusi, Tim Nawrot, Jan A Staessen, Tatiana Kuznetsova

**Affiliations:** KU Leuven Department of Cardiovascular Sciences, Research Unit Hypertension and Cardiovascular Epidemiology, University of Leuven, Leuven, Belgium; Hypertension Division, Department of Internal Medicine, University Clinical Centre Ljubljana, Ljubljana, Slovenia; Department of Health, University of Milano and Genomics and Bioinformatics Platform, Fondazione Filarete, Milano, Italy; Department of Medicine, University of Padova, Padova, Italy; MRC Unit for Lifelong Health and Ageing at University College London, London, UK; First Department of Cardiology, Interventional Electrocardiology and Hypertension, Jagiellonian University Medical College, Krakow, Poland; Institute of Internal and Preventive Medicine, Novosibirsk, Russian Federation; Novosibirsk State Medical University, Novosibirsk, Russian Federation; Department of Public Health, Occupational and Environmental Medicine, KU Leuven, Leuven, Belgium; Centre for Environmental Sciences, Hasselt University, Diepenbeek, Belgium; Department of Epidemiology, Maastricht University, Maastricht, Netherlands

**Keywords:** Epidemiology, Echocardiography, Diastolic function, *ATP12A*

## Abstract

**Background:**

Left ventricular (LV) function depends on the activity of transmembrane electrolyte transporters. Failing human myocardium has lower Na^+^/K^+^ ATPase expression and higher intracellular sodium concentrations. The *ATP12A* gene encodes a catalytic subunit of an ATPase that can function as a Na^+^/K^+^ pump. We, therefore, investigated the association between LV function and common genetic variants in *ATP12A*.

**Methods:**

A random sample of 1166 participants (53.7% women; mean age 49.5 years, 44.8% hypertensive) was recruited in Belgium, Poland, Italy and Russia. We measured transmitral early and late diastolic velocities (E and A) by pulsed wave Doppler, and mitral annular velocities (e’ and a’) by tissue Doppler. Using principal component analysis, we summarized 7 Doppler indexes – namely, E, A, e’ and a’ velocities, and their ratios (E/A, e’/a’, and E/e’) – into a single diastolic score. We genotyped 5 tag SNPs (rs963984, rs9553395, rs10507337, rs12872010, rs2071490) in *ATP12A.* In our analysis we focused on rs10507337 because it is located within a transcription factor binding site.

**Results:**

In the population-based analyses while adjusting for covariables and accounting for family clusters and country, rs10507337 C allele carriers had significantly higher E/A (*P* = 0.003), e’ (*P* = 5.8×10^−5^), e’/a’ (*P* = 0.003) and diastolic score (*P* = 0.0001) compared to TT homozygotes. Our findings were confirmed in the haplotype analysis and in the family-based analyses in 74 informative offspring.

**Conclusions:**

LV diastolic function as assessed by conventional and tissue Doppler indexes including a composite diastolic score was associated with genetic variation in *ATP12A*. Further experimental studies are necessary to clarify the role of *ATP12A* in myocardial relaxation.

**Electronic supplementary material:**

The online version of this article (doi:10.1186/s12881-014-0121-6) contains supplementary material, which is available to authorized users.

## Background

Left ventricular (LV) diastolic dysfunction refers to a condition in which abnormalities in LV function are present during diastole and is characterized by impaired relaxation and/or filling of the heart. LV diastolic dysfunction is associated with common risk factors such as hypertension and it can progress to symptomatic heart failure [1]. The prevalence of subclinical diastolic dysfunction in the general population increases with age and is as high as 25.1% [2-5]. Echocardiographic measurement of diastolic Doppler velocities of the transmitral blood flow (E and A peaks) and the mitral annular movement (e’ and a’ peaks) during early and late diastole allows the non-invasive assessment of LV diastolic function [6]. Moreover, low e’ velocity measured by Tissue Doppler Imaging (TDI) significantly and independently from other cardiovascular risk factors predicted higher risk of fatal and nonfatal cardiovascular events in patients with hypertension [7] or heart failure [8] and in general population [9]. Recently we demonstrated significant heritability of the diastolic Doppler indexes [10]. Understanding to what extent genetic factors along with anthropometric, hemodynamic factors and lifestyle influence diastolic Doppler indexes is an important issue in view of the relation of LV diastolic dysfunction with outcome.

Cardiomyocyte contraction and relaxation depend on the balance of electrolytes (calcium, sodium, potassium, etc.) across the cellular membranes [11,12]. The electrolyte gradients are maintained by transmembrane channels and adenosine triphosphate (ATP) dependent pumps. Experimental [13] and clinical studies [14] demonstrated that intracellular sodium concentration (Na^+^) is increased in failing cardiomyoctes as compared to normal myocardium. The possible cause for this observation might be related to changes in Na^+^/K^+^ ATPase expression and/or function [11,12]. Among the genes that encode the catalytic alpha subunit of Na^+^/K^+^-ATPase [15,16], *ATP12A* belongs to the family of P-type cation transport ATPases that can function as Na^+^/K^+^ ATPase in human cells [17]. Taking together, genetic variability in *ATP12A* might influence myocardial Na^+^ handling and consequently myocardial function. We recently genotyped top SNPs that might be associated with cardiovascular phenotypes and that have been identified in a recent genome-wide association study [18]. Among these SNPs common genetic variants in *ATP12A* were genotyped. Therefore, in the Flemish Study on Environment, Genes and Health Outcomes (FLEMENGHO) and the European Project On Genes in Hypertension (EPOGH) we investigated whether echocardiographic variables reflecting LV diastolic function are associated with common genetic variants in *ATP12A*.

## Methods

### Study participants

From August 1985 until December 2005, we randomly recruited a family-based population sample (the FLEMENGHO cohort) from a geographically defined area in northern Belgium as described in previous publications [4]. EPOGH recruited nuclear families from 1999 until 2001. The EPOGH investigators were trained at the Studies Coordinating Centre in Leuven, Belgium, and applied the same protocol, questionnaires and procedures, as used in FLEMENGHO [3]. All study participants provided a signed informed consent and the study was approved by the Ethical Committees of the University of Leuven, the University of Padova, Jagiellonian University Medical College, and the Novosibirsk Institute of Internal and Preventive Medicine. All clinical investigations were conducted according to the principles expressed in the Declaration of Helsinki. The initial response rate at enrolment was 75.0%.

In the FLEMENGHO study, from May 2005 until January 2010, we invited 1055 former participants from a previously identified random population for a follow-up examination at the field centre, including echocardiography. Of those, 828 renewed their written consent. In the EPOGH study, from January 2007 until September 2009, we invited 631 former participants for an examination including echocardiography. Of those, 561 gave their consent in writing. We excluded 223 subjects from analysis, because DNA was missing or of bad quality (*n* = 217), atrial fibrillation (*n* = 1) or because LV diastolic Doppler indexes could not be reliably measured (*n* = 5). Thus, 1166 subjects were analyzed including 777 FLEMENGHO participants (Noorderkempen, Belgium) and 389 EPOGH participants from Krakow, Poland (*n* = 143), Mirano, Italy (*n* = 109) and Novosibirsk, Russia (*n* = 137).

### Echocardiography

The participants refrained from smoking, heavy exercise, and drinking alcohol or caffeine-containing beverages for at least 3 hours before echocardiography.

#### Data acquisition

In each center one experienced physician did the ultrasound examination, using a Vivid7 Pro (GE Vingmed, Horten, Norway) interfaced with a 2.5- 3.5 MHz phased-array probe, according to a standardized protocol as published elsewhere [3,4]. With the subjects in partial left decubitus and breathing normally, the observer obtained images, together with a simultaneous ECG signal, from the parasternal long and short axes and from the apical 4- and 2-chamber long-axis views. M-mode echocardiograms of the LV were recorded from the parasternal long-axis view under control of the 2D image. The ultrasound beam was positioned just below the mitral valve at the level of the posterior chordae tendineae. To record mitral flow velocities from the apical window, we positioned the Doppler sample volume at the mitral valve tips.

Using TDI, the observer recorded low-velocity, high-intensity myocardial velocity at a high frame rate (>190 FPS), while adjusting the imaging angle to ensure parallel alignment of the ultrasound beam with the myocardial segment of interest. From the apical window, a 5 mm Doppler sample was placed at the septal, lateral, inferior and posterior sites of the mitral annulus to obtain the pulsed wave TDI velocities.

#### Off-line analysis

All echocardiographic recordings included at least 5 cardiac cycles and were digitally stored for off-line analysis. One experienced observer (TK) analyzed the digitally stored images from all centers, using the EchoPac software, version 4.0.4 (GE Vingmed, Horten, Norway), selecting and averaging 3 cardiac cycles obtained at the end-expiration. The observer was blinded to the genetic results. LV internal diameter and interventricular septal and posterior wall thickness were measured at end-diastole from the 2 dimensionally guided M-mode tracing according to the guidelines. When optimal orientation of M-mode ultrasound beam could not be obtained (in approximately 5% of echocardiographic recordings), the reader performed linear measurements on correctly oriented two-dimensional images. End-diastolic left ventricular dimensions were used to calculate LV mass by an anatomically validated formula. LV mass was indexed to body surface area (BSA). LV end-systolic and end-diastolic volumes were measured off-line using the standard biplane Simpson’s method.

We assessed LV diastolic function using recordings of conventional blood flow and tissue Doppler velocities. Pulsed-wave Doppler signals of transmitral blood flow were used to measure peak early (E) and late (A) diastolic velocities. From the pulsed wave TDI recordings, we measured the early (e’) and late (a’) peak diastolic velocities of the mitral annulus displacement, and the e’/a’ ratio at the 4 acquisition sites. We calculated the E/e’ ratio by dividing transmitral E peak by e’ averaged from the 4 acquisition sites. As reported previously [3], the inter-observer intra-session reproducibility across the four sampling sites ranged from 4.48% to 5.34% for e’ velocities and from 3.96% to 4.52% for a’ velocities.

### Other measurements

We administered a standardized questionnaire to collect detailed information on subjects’ medical history, smoking and drinking habits, and intake of medications. The conventional blood pressure was the average of five consecutive auscultatory readings obtained with the subject in the seated position. Hypertension was defined as a blood pressure of at least 140 mm Hg systolic or 90 mm Hg diastolic or as use of antihypertensive drugs. Body mass index was weight in kilograms divided by the square of height in meters.

### Genotypes

We extracted DNA from white blood cells. For genotyping of *ATP12A* SNPs we used 15 K Illumina Infinium custom chip (Illumina Inc, San Diego, CA), designed for the HYPERGENES project [18] to cover genes deemed to be relevant for hypertension and related target organ damage. Detailed information on the procedure of tag SNP selection for the custom chip is provided in Additional file 1. The human *ATP12A* gene lies within chromosome 13q12.1-q12.3 (http://www.ncbi.nlm.nih.gov/gene/479) and spans approximately 32 kb. It contains 23 exons and 22 introns [19]. The selected 8 tag SNPs were annotated according to the Genome Reference Consortium Human Build 37 hg19 (Additional file 1: Table S1). Three SNPs were excluded from the statistical analysis because of minor allele frequency (MAF) = 0.02 (rs2289909), genotyping call-rate <99% (rs1867767) or bad cluster according to Illumina Genome Studio (rs1001806, Additional file 1: Table S1). Of remaining 5 SNPs, rs963984, rs12872010 and rs2071490 lie within coding regions, but do not alter the amino acid sequence of the coded protein (synonymous). Two other SNPs (rs9553395 and rs10507337) are located in the 5′ flanking region, with rs10507337 within a transcription factor binding site.

### Statistical methods

For database management and statistical analysis, we used SAS software, version 9.3 (SAS Institute, Cary, NC). We compared means and proportions by the z test and by the χ^2^ test, respectively. We tested minimum allele frequency, proportion of missing genotypes and Hardy-Weinberg equilibrium using JMP Genomics, version 6.1 (SAS Institute, Cary, NC). We reconstructed haplotypes using the PROC HAPLOTYPE procedure implemented in the genetics module of the SAS software.

#### Principal component analysis

The diastolic Doppler velocities are measured using similar technique (Doppler) and are highly intra-correlated. Therefore, some redundancy in the cumulative variance of the Doppler velocities exists. This redundancy can be decreased by a mathematical procedure, the principal component analysis. This thechnique enables summarization of intra-correlated variables into an artificial score that carries information from all the contributing variables. Individual values of the artificial score are calculated by summarization of optimally weighted values of the contributing measured indexes. We, therefore, summarized transmitral E and A blood flow velocities, averaged TDI e’ and a’ mitral annulus velocities and their ratios (E/A, e’/a’, E/e’) into a single composite diastolic score. Normality of diastolic score distribution was evaluated by Shapiro-Wilk’s statistic and skewness by computation of the coefficient of skewness. The association between the composite diastolic score and velocities was described using factors loadings. We performed stepwise linear regression to identify correlates of the composite diastolic score with sex, age, height, weight, body mass index, heart rate, systolic and diastolic blood pressures, left ventricular mass index, and ejection fraction. We set the *P* value for variables to enter and stay in the regression models at 0.10.

#### Population-based analysis

First, we performed the association analyses of dependent variables (LV phenotypes) with the genotypes or haplotypes of interest by use of a mixed model in each examination center. Covariables with known relevance for LV structure and function [3] were included in the models as fixed effects, while family clusters was modeled as a random effect. Then we performed the association analyses in all centers combined while examination centers were modeled as random effects. We tested whether observed effect sizes are homogeneous across examination centers. The heterogeneity test statistic (Cochran’s Q) was computed by summing the squared deviations of each center estimate from the overall meta-analytic estimate, weighting each center contribution in the same manner as in the meta-analysis. We also reported the *P*-values corrected for multiple testing using the Bonferroni method.

#### Family-based analysis

We performed the transmission disequilibrium test for quantitative traits (QTDT). We evaluated the within- and between-family components of phenotypic variance using the orthogonal model as implemented by Abecasis *et al.* [20] in the QTDT software (version 2.6.1; http://www.sph.umich.edu/csg/abecasis/QTDT). In this model the between-family component is sensitive to population structure whereas the within-family component is significant in the presence of transmission disequilibrium.

## Results

### Characteristics of participants by center, *ATP12A* alleles and genotypes and haplotypes frequencies

Table 1 summarizes baseline characteristics of participants by examination center. Overall, the 1166 white European participants included 626 (53.7%) women and 522 (44.8%) hypertensive patients of whom 315 (27.0%) were on antihypertensive drug treatment. Mean age (±SD) was 49.5 ± 15.2 years. Polish participants were younger, had higher systolic blood pressure and less frequently reported a daily alcohol consumption of ≥5 gram than participants from other centers (Table 1). Composite diastolic Doppler score were highest in the Polish center compared to the remaining centers (Table 1). Additional file 1: Table S2 presents baseline characteristics of participants by sex.

**Table 1:** **Characteristics of participants by country**

	**Belgium**	**Poland**	**Italy**	**Russia**
Characteristic	(*n* = 777)	(*n* = 143)	(*n* = 109)	(*n* = 137)
*Anthropometrics*				
Female sex, *n* (%)	399 (51.4)	78 (54.6)	60 (55.1)	89 (65.9)*
Age (years)	51.0 ± 15.5	42.9 ± 13.8*	50.3 ± 13.5†	47.7 ± 14.5*†
Height (cm)	168.7 ± 9.4	169.5 ± 9.1	167.0 ± 9.0†	167.1 ± 9.6†
Weight (kg)	75.5 ± 14.4	78.1 ± 15.1	75.2 ± 14.2	77.3 ± 16.2
Body mass index (kg/m^2^)	26.5 ± 4.3	27.2 ± 5.3	26.9 ± 4.6	27.7 ± 5.6*
Systolic pressure (mmHg)	129.3 ± 17.6	137.7 ± 18.9*	125.4 ± 17.1*†	132.4 ± 23.8‡
Diastolic pressure (mmHg)	79.6 ± 9.4	84.2 ± 11.7*	82.3 ± 10.1*	84.4 ± 14.1*
Heart rate (beats/minute)	61.0 ± 9.7	66.4 ± 11.2*	65.9 ± 10.4*	66.2 ± 10.3*
*Questionnaire data*				
Current smoking, *n* (%)	161 (20.7)	31 (21.7)	18 (16.5)	35 (25.6)
Drinking alcohol, *n* (%)	309 (39.8)	17 (11.9)*	53 (48.6)†	38 (27.7)†‡
Hypertensive, *n* (%)	323 (41.6)	81 (56.6)*	51 (46.8)	67 (48.9)
Treated for hypertension, *n* (%)	197 (25.4)	44 (32.8)	25 (22.9)	49 (35.8)*‡
*Conventional echocardiography*				
LV internal diameter (cm)	5.04 ± 0.47	5.06 ± 0.47	4.91 ± 0.44*†	4.99 ± 0.41
Interventricular septum (cm)	0.98 ± 0.17	0.96 ± 0.15	0.94 ± 0.15*	0.98 ± 0.18
Posterior wall (cm)	0.90 ± 0.14	0.90 ± 0.13	0.88 ± 0.13	0.90 ± 0.13
LV mass index (g/m^2^)	92.4 ± 22.0	90.8 ± 20.9	86.0 ± 18.6*	91.2 ± 21.9
Ejection fraction (%)	63.3 ± 6.75	63.9 ± 6.56	62.6 ± 6.15	64.1 ± 6.13
*Diastolic function*				
Transmitral E peak (cm/s)	75.5 ± 16.1	74.5 ± 14.9	68.8 ± 14.9*†	67.0 ± 14.5*†
Transmitral A peak (cm/s)	65.0 ± 17.4	58.6 ± 14.7*	59.6 ± 19.5*	61.0 ± 18.0*
Transmitral E/A ratio	1.26 ± 0.48	1.36 ± 0.44*	1.28 ± 0.49	1.21 ± 0.49†
TDI e’ peak (cm/s)	11.4 ± 3.65	11.3 ± 3.51	10.3 ± 3.15*†	10.4 ± 3.40*
TDI a’ peak (cm/s)	10.1 ± 2.11	9.08 ± 1.93*	10.2 ± 2.12†	9.33 ± 2.05*‡
e’/a’ ratio	1.26 ± 0.71	1.36 ± 0.64	1.07 ± 0.52*†	1.29 ± 0.68‡
E/e’ ratio	7.10 ± 2.19	7.10 ± 2.16	7.15 ± 2.26	7.04 ± 2.63
Composite diastolic score	−0.028 ± 2.11	0.41 ± 1.93*	−0.27 ± 1.92†	−0.081 ± 2.07†

Values are mean ± SD or number of subjects (%). LA, LV and TDI indicate left atrium, left ventricle and Tissue Doppler Imaging. Significance for between-country differences: *P ≤ 0.05 vs Belgium; †*P* ≤ 0.05 vs Poland; ‡*P* ≤ 0.05 vs Italy.

Additional file 1: Table S1 lists the chromosome position, location type, allele frequencies, call frequencies and tagged SNPs of the 8 selected *ATP12A* SNPs. In our analyses we focused on rs10507337 because it is located within a transcription factor binding site and, therefore, it might influence the *ATP12A* gene expression. The r^2^ reflecting linkage disequilibrium between rs10507337 and rs12872010 was 0.83. On the other hand, we observed no correlation between rs10507337 and three other *ATP12A* SNPs, rs963984, rs9553395 and rs2071490, with the r^2^ values ranged from 0.008 to 0.245, respectively. The genotype frequencies of rs10507337 complied with Hardy-Weinberg equilibrium (*P* = 0.97). Center specific and cumulative rs10507337 genotype distributions are listed in Table 2. As there were only 5 (0.4%) rs10507337 C allele homozygotes (all from the Belgian center), we contrasted rs10507337 C allele carriers with TT homozygotes and, therefore, use a recessive association model. Additional file 1: Table S3 lists the clinical and echocardiographic characteristics of the participants by rs10507337 genotypes. In unadjusted analysis, rs10507337 C allele carriers had lower heart rate, higher transmitral E, E/A ratio, TDI e’ and e’/a’ ratio compared to TT homozygotes. There were no other differences in clinical or echocardiographic parameters between these two groups.

**Table 2:** **rs10507337 genotype distribution by country**

**Country**	**Genotype**		
	**TT N (%)**	**TC N (%)**	**CC N (%)**
Belgium (*n* = 777)	663 (85.3)	109 (14.0)	5 (0.64)
Italy (*n* = 109)	101 (92.7)	8 (7.34)	/
Poland (*n* = 143)	123 (86.0)	20 (14.0)	/
Russia (*n* = 137)	130 (94.9)	7 (5.11)	/
All participants (*n* = 1166)	1017 (87.2)	144 (12.4)	5 (0.43)

We used the selected 5 tag SNPs of *ATP12A* (rs9553395, rs10507337, rs2071490, rs12872010 and rs963984; Additional file 1: Table S1) to reconstruct haplotypes. Haplotype frequencies for TTTCC, TTCCA, TTCCC, CTTCC, CTCCA, CCTTC were 67.4%, 7.1%, 8.5%, 6.9%, 2.3% and 5.3%, respectively.

### Composite diastolic score

**Figure 1:**
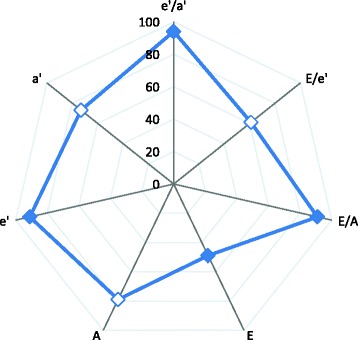
**Loading of composite diastolic Doppler score.** Absolute values of bivariate correlations (factor loadings) between the composite diastolic score and each of the diastolic parameters. The correlations of transmitral A, TDI a’ and E/e’ ratio to the composite score were negative and are represented by non-filled points.

Because of the high intra-correlation of Doppler diastolic velocities, we summarized these traits into a single diastolic score using principal component analysis. The first principal component accounted for 61.5% of the overall variance in the 7 contributing Doppler indexes. Therefore, we used the first principal component as a normally distributed summary score (mean 0; standard deviation 1; Additional file 1: Figure S2). Figure 1 shows factor loadings for each of the diastolic Doppler measures which we used to calculate the composite diastolic score. Participants with better diastolic function profile (higher transmitral *and* tissue Doppler velocities during early diastole *and* lower E/e’) had higher values of the composite score. In stepwise multiple regression, the composite diastolic score significantly and independently decreased with age, body mass index, heart rate, diastolic blood pressure and left ventricular mass index (Table 3). The explained variance totaled 79.2%.

**Table 3:** **Correlates of the composite diastolic Doppler score selected in stepwise regression**

**Parameter**	**Diastolic Doppler score**			
	**Partial R** ^**2**^ **(%)**	**Parameter estimate ± SE**	**95%CI**	***P*** **-value**
Age (+10 years)	66.0	−1.01 ± 0.021	−1.06 to −0.98	<0.0001
Body mass index (+1 kg/m^2^)	4.37	−0.080 ± 0.0068	−0.093 to −0.066	<0.0001
Heart rate (+10 beats/minute)	7.49	−0.47 ± 0.029	−0.52 to −0.40	<0.0001
Diastolic pressure (+10 mmHg)	1.08	−0.21 ± 0.029	−0.26 to −0.15	<0.0001
LVMI (+10 g/m^2^)	0.18	−0.048 ± 0.015	−0.077 to −0.018	0.0016
Total R^2^ (%)	79.2			

Values are mutually adjusted partial regression coefficients ± SE. CI, confidence interval; LVMI, left ventricle mass index.

### Population-based association study

We adjusted the model for important covariables such as sex, age, body mass index, heart rate and diastolic blood pressure as well as for non-independence of observations within families and centers. Table 4 lists the adjusted Doppler diastolic indexes and composite score by rs10507337 genotype by country and in all centers combined. When all centers were pooled (Table 4), subjects carrying at least one rs10507337 C allele had significantly higher values of TDI e’ (*P* = 5.8×10^−5^), transmitral E/A (*P* = 0.003), TDI e’/a’ (*P* = 0.003) and composite diastolic score (*P* = 0.0001). The heterogeneity tests (*P* = 0.04) suggested that there was borderline but significant heterogeneity among the centers for peaks E and e’ , e’/a’ ratio and composite diastolic score (Table 4). After excluding from the analyses the Polish participants, who were younger than the participants from other centers, we did not observe a heterogeneity among the remaining centers (*P* > 0.40). Haplotype analysis further confirmed that in all subjects combined, composite diastolic score (*P* = 0.001; Table 5) and related Doppler parameters such as TDI e’, E/A, e’/a’ (*P* ≤ 0.009; Table 6) were significantly higher in CCTTC carriers than in noncarriers. This is the only haplotype that included rs10507337 C allele.

**Table 4:** **Adjusted Doppler diastolic indexes and composite score by rs10507337 by country and all centers combined**

**Country**	**Genotype**	**LV diastolic indexes adjusted means ± SE**							
		**E peak (cm/s)**	**A peak (cm/s)**	**E/A ratio**	**TDI e’ (cm/s)**	**TDI a’ (cm/s)**	**e’/a’ ratio**	**E/e’ ratio**	**Composite diastolic score**
Belgium (*n* = 777)	TT (*n* = 663)	75.2 ± 0.58	65.3 ± 0.51	1.25 ± 0.013	11.3 ± 0.092	10.2 ± 0.084	1.23 ± 0.018	7.13 ± 0.064	−0.097 ± 0.046
	C allele carriers (*n* = 114)	76.9 ± 1.32	62.9 ± 1.12	1.34 ± 0.028	12.2 ± 0.19	9.99 ± 0.16	1.38 ± 0.038	6.87 ± 0.16	0.35 ± 0.094
	*P*	0.22	0.044	0.0008	8.2×10^−6^	0.15	0.0002	0.12	3.5×10^−6^
Italy (*n* = 109)	TT (*n* = 101)	67.7 ± 1.68	59.3 ± 1.32	1.27 ± 0.030	10.3 ± 0.19	10.2 ± 0.17	1.05 ± 0.035	7.11 ± 0.20	−0.30 ± 0.10
	C allele carriers (*n* = 8)	81.2 ± 4.80	59.1 ± 4.74	1.38 ± 0.11	11.6 ± 0.62	9.87 ± 0.58	1.18 ± 0.11	7.30 ± 0.66	0.26 ± 0.34
	*P*	0.006	0.97	0.34	0.042	0.58	0.26	0.78	0.12
Poland (*n* = 143)	TT (*n* = 123)	74.4 ± 1.30	58.4 ± 1.06	1.36 ± 0.025	11.4 ± 0.20	9.13 ± 0.15	1.37 ± 0.030	7.05 ± 0.18	0.43 ± 0.087
	C allele carriers (*n* = 20)	72.0 ± 3.31	58.0 ± 2.60	1.34 ± 0.066	10.8 ± 0.51	9.11 ± 0.38	1.24 ± 0.080	7.16 ± 0.43	0.17 ± 0.22
	*P*	0.50	0.87	0.80	0.27	0.95	0.14	0.80	0.27
Russia (*n* = 137)	TT (*n* = 130)	65.6 ± 1.24	59.8 ± 1.03	1.20 ± 0.03	10.3 ± 0.19	9.45 ± 0.15	1.24 ± 0.044	6.96 ± 0.20	−0.14 ± 0.11
	C allele carriers (*n* = 7)	61.8 ± 4.86	59.1 ± 3.92	1.22 ± 0.12	10.4 ± 0.69	9.74 ± 0.57	1.32 ± 0.16	6.20 ± 0.78	0.012 ± 0.38
	*P*	0.45	0.86	0.84	0.93	0.61	0.62	0.34	0.70
All participants (*n* = 1166)	TT (*n* = 1017)	70.9 ± 1.74	60.8 ± 1.50	1.27 ± 0.016	10.68 ± 0.25	9.66 ± 0.24	1.22 ± 0.028	7.15 ± 0.11	−0.050 ± 0.036
	C allele carriers (*n* = 149)	72.9 ± 2.06	59.4 ± 1.76	1.34 ± 0.028	11.36 ± 0.29	9.52 ± 0.27	1.33 ± 0.042	6.98 ± 0.18	0.28 ± 0.083
	*P*	0.11	0.19	0.003	5.8×10^−5^	0.32	0.003	0.28	0.0001
	P*	/	/	0.014	0.0007	/	0.007	/	0.0008
	*P* for heterogeneity	0.04	0.81	0.48	0.04	0.79	0.04	0.66	0.04

Values are least square means ± SE adjusted for family clusters, country (in combined analysis), sex, age, body mass index, diastolic blood pressure and heart rate. TDI indicates Tissue Doppler Imaging, SE, standard error. *P*-values are for the differences between rs10507337 TT homozygotes and C allele carriers. P*-values are for the differences after Bonferroni adjustment for multiple comparisons.

**Table 5:** **Composite diastolic Doppler score by ATP12A haplotypes in all centers combined**

	**Number of coded haplotypes**				
**Haplotype**	**00**	**01**	**11**	***P***	P*
*TTTCC*					
N (%)	104 (9.0)	552 (47.3)	510 (43.7)		
Adjusted	0.19 ± 0.10	−0.012 ± 0.045	−0.057 ± 0.048	0.07	/
*TTCCA*					
N (%)	1005 (86.2)	157 (13.5)	4 (0.34)		
Adjusted	−0.020 ± 0.037	0.042 ± 0.078		0.45	/
*TTCCC*					
N (%)	972 (83.4)	184 (15.8)	10 (0.86)		
Adjusted	0.005 ± 0.037	−0.080 ± 0.072		0.27	/
*CTTCC*					
N (%)	1009 (86.5)	154 (13.2)	3 (0.26)		
Adjusted	−0.019 ± 0.038	0.041 ± 0.079		0.47	/
*CTCCA*					
No. (%)	1113 (95.5)	53 (4.55)	/		
Adjusted	−0.005 ± 0.035	−0.12 ± 0.13		0.41	/
*CCTTC*					
No. (%)	1047 (89.8)	115 (9.86)	4 (0.34)		
Adjusted	−0.039 ± 0.036	0.27 ± 0.092		0.001	0.003

Values are least square means ± SE adjusted for family clusters, country, sex, age, body mass index, diastolic blood pressure and heart rate. P*-values are for the differences after Bonferroni adjustment for multiple comparisons.

**Table 6:** **Adjusted Doppler diastolic indexes and composite score by CCTTC haplotype in all centers combined**

	**CCTTC**		***P***
**LV diastolic indexes**	**00 (n = 1047)**	**01 or 11 (n = 119)**	
Transmitral E peak (cm/s)	71.0 ± 1.75	72.3 ± 2.15	0.33
Transmitral A peak (cm/s)	60.7 ± 1.50	59.5 ± 1.83	0.26
Transmitral E/A ratio	1.27 ± 0.016	1.34 ± 0.031	0.009
TDI e’ peak (cm/s)	10.7 ± 0.25	11.3 ± 0.30	0.0006
TDI a’ peak (cm/s)	9.65 ± 0.24	9.53 ± 0.28	0.42
e’/a’ ratio	1.23 ± 0.028	1.33 ± 0.045	0.008
E/e’ ratio	7.15 ± 0.11	6.97 ± 0.19	0.29
Composite diastolic score	−0.039 ± 0.036	0.27 ± 0.092	0.001

Values are least square means ± SE adjusted for family clusters, country, sex, age, body mass index, diastolic blood pressure and heart rate. *P*-values are for the differences between CCTTC carriers and the rest of *ATP12A* genotypes.

Our findings remained consistent after exclusion of subjects with history of coronary heart disease or valve abnormalities (n = 67; Additional file 1: Table S4) or subjects on antihypertensive drugs (n = 315; Additional file 1: Table S5). For other LV phenotypes (Additional file 1: Table S6), none of the phenotype-genotype associations reached statistical significance. With adjustment for multiple testing applied, we observed that TDI e’, e’/a’ ratio and composite diastolic score were significantly (*P* ≤ 0.008) higher in carriers of rs12872010 T allele compared to non-carriers (Additional file 1: Table S7). These associations were somewhat expected given the high correlation between *ATP12A* rs10507337 and rs12872010. We did not observe any significant associations with other tested SNPs (Additional file 1: Tables S8, S9 and S10) or haplotypes (Table 5).

### Family-based association study

Our study population (*n* = 1166) included 749 founders and 417 offspring from 357 families. In our family-based analyses we used 74 informative offspring belonging to 35 families (mean age 34.6 ± 8.2 years; 56.9% women). The number of offspring per informative family amounted to one in 10 families, two in 17 families, three in 4 families, and more than three in 4 families. We adjusted the QTDT analyses as described in the previous section. For LV diastolic function indexes in relation to rs10507337 genotypes, the orthogonal model did not demonstrate population stratification (*P* ≥ 0.08). Transmitral E/A, TDI e’ , TDI e’/a’ and composite diastolic score significantly increased with transmission of at least one C allele to offspring (Table 7). The effect sizes of the within-family components averaged 0.17 (χ^2^ = 6.61, *P* = 0.010) for E/A, 1.19 cm/s (χ^2^ = 8.61, *P* = 0.003) for e’, 0.26 (χ^2^ = 9.13, *P* = 0.003) for e’/a’ and 0.62 (χ^2^ = 9.04, *P* = 0.003) for composite score (Table 7).

**Table 7:** **Results of the QTDT analyses for the rs10507337 C allele in relation to Doppler diastolic indexes and composite score**

	**Parameter estimate**	**χ** ^**2**^	***P***
**LV diastolic indexes**			
Transmitral E peak (cm/s)	1.40	0.22	0.64
Transmitral A peak (cm/s)	−3.30	1.59	0.21
Transmitral E/A ratio	0.17	6.61	0.010
TDI e’ peak (cm/s)	1.19	8.61	0.003
TDI a’ peak (cm/s)	−0.25	0.21	0.65
TDI e’/a’ ratio	0.26	9.13	0.003
E/e’ ratio	−0.33	0.74	0.39
Composite diastolic score	0.62	9.04	0.003

The orthogonal model accounted for between- and within-family variability components. The parameter estimate for the within-family component of the rs10507337 C allele indicates the direction and size of the association. Analyses were adjusted for center, sex, age, body mass index, heart rate and diastolic blood pressure. TDI, Tissue Doppler Imaging.

## Discussion

The main finding of the present study was that LV diastolic function as assessed by conventional and tissue Doppler indexes was associated with genetic variation in the *ATP12A* promoter. Carriers of rs10507337 C allele had better pattern of myocardial relaxation as compared to non-carriers. Because rs10507337 C allele occurred only in one reconstructed haplotype (CCTTC), we found also the significant associations between this haplotype and LV diastolic function indexes. The family-based analyses included only 74 informative offspring but nevertheless confirmed that transmission of at least one C allele to offspring was associated with higher transmitral and tissue Doppler velocities during early diastole and composite diastolic score. On the contrary, we did not observe any association of the genetic variants in *ATP12A* and other LV phenotypes including LV mass index and ejection fraction.

The gold standard for assessing diastolic function remains the pressure-volume relationship, but this requires an invasive approach. Doppler measurements of mitral inflow and the TDI technique open up the possibility of evaluating non-invasively diastolic function [6]. In our study, we assessed LV diastolic function non-invasively using the transmitral flow and the TDI mitral annular velocities. Previous studies validated these indexes versus invasive measures of diastolic function [6]. LV diastolic dysfunction is defined as functional abnormalities that exist during LV relaxation and filling. Impaired myocardial relaxation is characterized by decreased early (E peak), but enhanced atrial LV filling (A peak) as well as less vigorous mitral annulus motion during early diastole (TDI e’ peak). Moreover, e’ peak velocity along the LV longitudinal axis is less susceptible to the effects of an increased preload and therefore provides a more direct measure of myocardial relaxation than, for instance, the transmitral E peak velocity. In addition, combining transmitral flow velocity with annular velocity (E/e’ ratio) might be a tool for assessing the LV filling pressure, which combines the influence of the transmitral driving pressure and myocardial relaxation [6].

To our knowledge, no previous study reported the association of cardiovascular phenotypes with the *ATP12A* gene. So far, only a whole DNA array survey in spontaneously hypertensive rats identified the *ATP12A* gene among probable candidate genes for hypertension [21]. Kinoshita *et al.* [21] demonstrated that lower *ATP12A* expression might contribute to development of hypertension in spontaneously hypertensive rats. There is a high level of structural similarity (up to 86%) in the *ATP12A* gene between humans and rodents [16]. The *ATP12A* gene product is a catalytic alpha (ATP12Aα) subunit of an ATP dependent transmembrane electrolyte pump family (X^+^-K^+^-ATPases) [16,19] which is expressed in human myocardium (http://www.genecards.org/cgi-bin/carddisp.pl?gene=ATP12A). Functional X^+^-K^+^-ATPases are heterodimers, composed of alpha and beta subunits. Alpha subunits of ATPases have catalytic and ion-binding properties [22]. Previous studies in Xenopus oocytes and human HEK293 cells demonstrated that ATP12Aα could be assembled to ATPase that functions as a Na^+^-K^+^-pump [17,23]. Moreover, ATP12Aα is sensitive to cardiac glycoside ouabain, specific inhibitor of Na^+^-K^+^-ATPases [24].

Small perturbations in Na^+^ concentration due to changes of Na^+^-K^+^-ATPase expression and activity, might lead to important changes of Ca^2+^ extrusion, a process central for myocardial contractility and relaxation [11,12]. Schwinger *et al.* [25,26] demonstrated that failing human myocardium had low level of Na^+^-K^+^-ATPase concentration and, a consequence, had elevated intracellular Na^+^ levels [12,13]. Moreover, studies in rodent hearts showed that decrease in ATP production effects Na^+^-K^+^-ATPase activity and leads to elevated intracellular Na^+^ concentration [27].

Involvement of Na^+^-K^+^-ATPase in electrolyte homeostasis makes *ATP12A* a possible candidate gene that might modify cardiac function. In our study, we investigated the genetic variation which is located in a transcription factor binding site of the *ATP12A* promoter area. Several transcription factors can bind this promoter region including CTCF (CCCTC-binding factor) and NF-kB that both act in NF-kB cascade [19]. Of notice, levels of NF-kB were increased in failing human myocardium [28]. Therefore, in conditions related to disturbances in ATP production such as ischemia or heart failure, these transcription factors might modify expression of Na^+^-K^+^-ATPase.

In our study we observed that subjects with minor C allele in the *ATP12A* promoter area had significantly higher transmitral and tissue Doppler velocities during early diastole and, therefore, demonstrated an enhanced early myocardial relaxation compared to overall population mean. Overall, these findings might be indicative of the functional importance of the described genetic variation in *ATP12A.* However, we do not know whether rs10507337 C allele is associated with increased or decreased expression of the *ATP12A* gene. The precise mechanisms underlying the association of LV diastolic function with *ATP12A* remain to be elucidated.

Our echocardiographic estimation of diastolic function included measurement of diastolic blood flow (A, E) and mitral annular movement (a’, e’), and calculation of several ratios (E/A, e’/a’, E/e’). Since each of these Doppler indexes represents a somewhat distinct feature of diastolic function, none of them is sufficient to stand alone [1]. On the other hand, because diastolic Doppler indexes are measuring the same construct, e.g. velocities of transmitral blood flow and LV mitral annulus movement during diastole and its ratios, it is possible to reduce the measured phenotypes into a principal component (composite score) that will account for most of the variance in the variables. Indeed, all used diastolic indexes are intra-correlated and, therefore, some redundancy is expected. This continuously and normally distributed diastolic Doppler score accounted for 61.5% of the variance in the contributing indexes. In our study, subjects with better diastolic function had higher values of the composite score. Similarly, the composite diastolic score was significantly higher in rs10507337 C allele carriers and in CCTTC haplotype carriers. Because of the linkage disequilibrium between rs10507337 and rs12872010, this association was also observed in rs12872010 minor allele carriers. Hence, our findings remained consistent and we may conclude that the composite score of diastolic Doppler velocities might be used in genomic studies as a phenotype describing LV diastolic function.

The present study must be interpreted within the context of its limitations and strengths. All participants were white Europeans. Thus, the association cannot be generalized to other ethnic or racial groups. Transmitral and TDI diastolic velocities are quantitative echocardiographic traits that arise from complex interaction between multiple genes, hemodynamic, and environmental factors and are prone to measurement error. For the present study, one experienced observer in each center performed all echocardiographic examinations. Digitally stored images were post-processed centrally by one observer with high reproducibility [4]. There was also a high degree of internal consistency between the results of the population-based and family-based analyses. The between-family components for rs10507337 were not statistically significant, which makes it unlikely that our results are driven by population stratification.

## Conclusion

Left ventricular diastolic function as assessed by conventional and tissue Doppler indexes was associated with genetic variation in the *ATP12A* promoter. We observed that carriers of rs10507337 C allele or CCTTC haplotype had better pattern of myocardial relaxation as compared to non-carriers. Further studies are necessary to clarify the functional significance of this genetic variation.

## Additional file

Additional file 1:
**a: Supplemental methods.** b: **Figure S1.** Local plot of ATP12A region mapped by selected SNPs. c: **Figure S2.** Distribution and probability plot for the first principal component Describing LV diastolic function. d: **Table S1.** Selected SNPs for ATP12A. e: **Table S2.** Characteristics of participants by sex. f: **Table S3.** Characteristics of participants by rs10507337 genotypes. g: **Table S4.** Adjusted Doppler diastolic indexes and composite score by ATP12A rs10507337 in subjects without a previous history of coronary heart disease or valve abnormalities. h: **Table S5.** Adjusted Doppler diastolic indexes and composite score by ATP12A rs10507337 in untreated subjects. i: **Table S6.** Adjusted LV echocardiographic phenotypes by rs10507337. j: **Table S7.** Adjusted Doppler diastolic indexes and composite score by rs12872010 by country and in all centres combined. k: **Table S8.** Adjusted Doppler diastolic indexes and composite score by rs9553395 by country and in all centres combined. l: **Table S9.** Adjusted Doppler diastolic indexes and composite score by rs2071490 by country and in all centres combined. m: **Table S10.** Adjusted Doppler diastolic indexes and composite score by rs963984 by country and in all centres combined.

## Abbreviations

A: the transmitral peak late diastolic velocity
a’: the peak late diastolic mitral annular velocity
ATP: Adenosine triphosphate
*ATP12A*: Gene that encode the catalytic alpha subunit of Na^+^/K^+^-ATPase
BSA: Body surface area
E: the transmitral peak early diastolic velocity
E/A ratio: E velocity/A velocity ratio
e’: the peak early diastolic mitral annular velocity
e’/a’ ratio: e’ velocity/a’ velocity ratio
E/e’ ratio: E velocity/e’ velocity ratio
EPOGH: European Project on Genes in Hypertension
EF: Ejection fraction
FLEMENGHO: Flemish Study on Environment, Genes and Health Outcomes
LV: Left ventricle
MAF: Minor allele frequency
QTDT: Quantitative transmission disequilibrium test
SD: Standard deviation
TDI: Tissue Doppler Imaging
