# Supplementary material for: Left ventricular diastolic function associated with common genetic variation in ATP12A in a general population
Source: BMC Med Genet. 2014 Nov 4;15:121. doi: 10.1186/s12881-014-0121-6 (PMC4411923; doi:10.1186/s12881-014-0121-6)
Supplement: Additional file 1: — a: Supplemental methods. b: Figure S1. Local plot of ATP12A region mapped by selected SNPs. c: Figure S2. Distribution and probability plot for the first principal component Describing LV diastolic function. d: Table S1. Selected SNPs for ATP12A. e: Table S2. Characteristics of participants by sex. f: Table S3. Characteristics of participants by rs10507337 genotypes. g: Table S4. Adjusted Doppler diastolic indexes and composite score by ATP12A rs10507337 in subjects without a previous history of coronary heart disease or valve abnormalities. h: Table S5. Adjusted Doppler diastolic indexes and composite score by ATP12A rs10507337 in untreated subjects. i: Table S6. Adjusted LV echocardiographic phenotypes by rs10507337. j: Table S7. Adjusted Doppler diastolic indexes and composite score by rs12872010 by country and in all centres combined. k: Table S8. Adjusted Doppler diastolic indexes and composite score by rs9553395 by country and in all centres combined. l: Table S9. Adjusted Doppler diastolic indexes and composite score by rs2071490 by country and in all centres combined. m: Table S10. Adjusted Doppler diastolic indexes and composite score by rs963984 by country and in all centres combined. [file 12881_2014_121_MOESM1_ESM.docx]

**Left ventricular diastolic function associated with common genetic variation in *ATP12A* in a general population**Short title: LV diastolic function and *ATP12A*

**Supplemental Material**

**Supplemental Methods**

**SNPs selection**

For genotyping of *ATP12A* SNPs we used 15K Illumina Infinium custom chip (Illumina Inc, San Diego, CA), designed for the HYPERGENES^1^ project to fine map genes deemed to be relevant for hypertension and related target organ damage. In order to fine map these genes, we selected about 15000 SNPs for a custom iSelect HD Illumina using the following procedure. First, we extracted all markers in the relevant genes mapped by the best SNPs using the Illumina Gene Annotation of 1M duo and 1M quad chips as reference (the densest Illumina chips available). Then, we excluded the SNPs mapping more than 100kb upstream and downstream of the gene-start and gene-end. We also excluded triallelic and quadriallelic markers or SNPs that did not univocally map in the genome. After that we chose SNPs with HapMap minor allele frequency (MAF) ≥0.01 in at least one of the three populations of the confirmation sample: CEU, CHB and YRI. Finally, we selected the best tag SNPs which are representative in a region of the genome with high linkage disequilibrium that allow to identify genetic variation without genotyping every SNP in the region.

As shown in Table S1 and Figure S1, the *ATP12A* gene was fine-mapped by 8 SNPs that allow reconstructing the genotypes of about 90 SNPs. In particular, they cover a genomic area between two recombination peaks mapping the 5’-flanking and the intronic/coding region of the gene but not the 3’-flanking area.

**Reference**

**(1)**  Salvi E, Kutalik Z, Glorioso N et al. Genomewide association study using a high-density single nucleotide polymorphism array and case-control design identifies a novel essential hypertension susceptibility locus in the promoter region of endothelial NO synthase. *Hypertension* 2012;59:248-255.

**Figure S1**

**Local Plot of *ATP12A* Region Mapped by Selected SNPs.** The horizontal axis represents the physical position on the chromosome (build 37, hg19), the vertical axis and the blue line indicate the recombination rate. Genotyped SNPs are highlighted in red and tagged SNPs are represented with a vertical line.

**
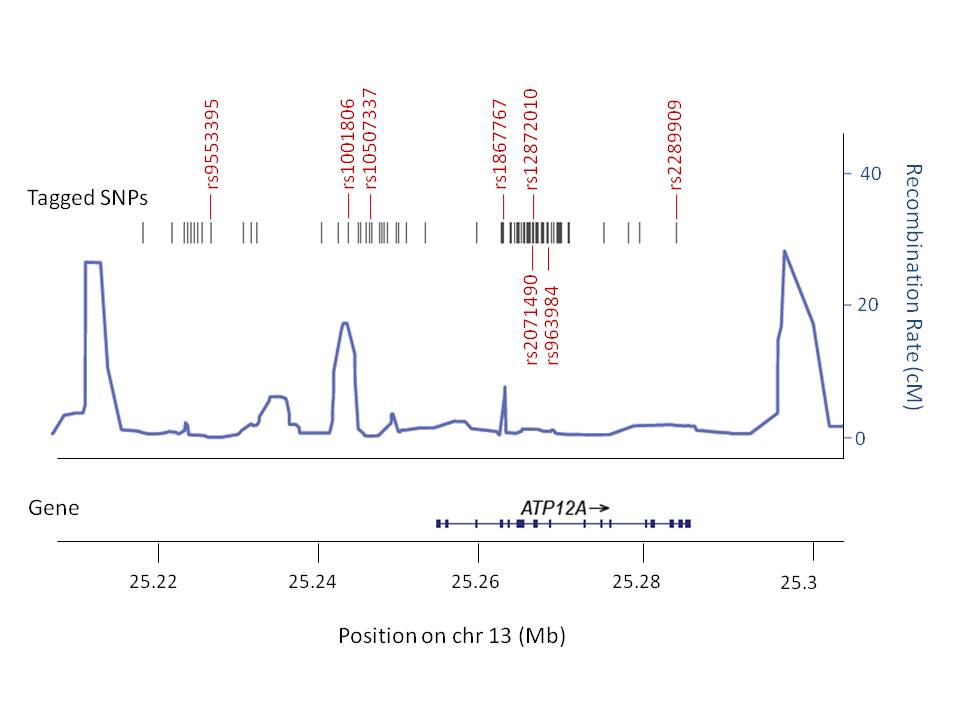
**

**
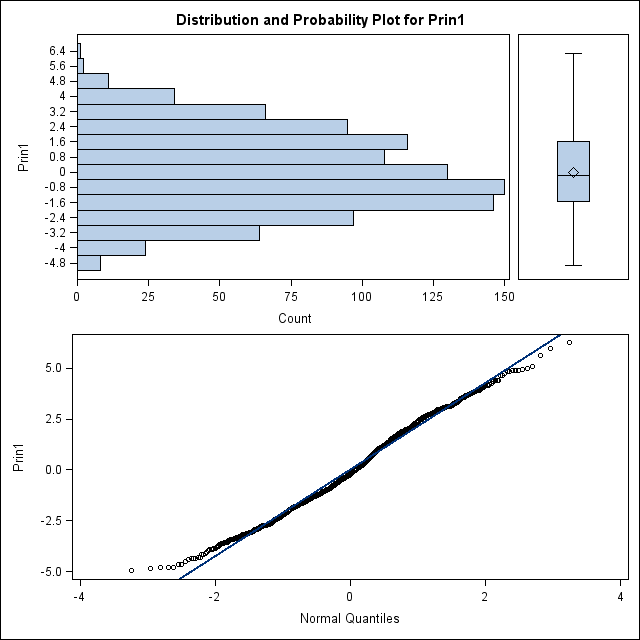
Figure S2**

**Distribution and Probability Plot for the First Principal Component Describing LV Diastolic Function.**

**Table S1 Selected SNPs for *ATP12A***

| **SNP ID** | **Position (bp)** | **Location**  **type** | **Minor/major**  **Alleles** | **MAF** | **Call Frequency** | **Tagged SNPs** |
| --- | --- | --- | --- | --- | --- | --- |
| rs9553395 | 25225837 | 5’-flanking | C/T | 0.152 | 0.996 | rs9553394,rs751853,rs12873598,rs9553392,rs2902463,rs9551133,rs9553397, rs9553387, rs34123791,rs9551134,rs7988031 |
| rs1001806 | 25242931 | 5’-flanking | T/C | Bad cluster | Bad cluster | / |
| rs10507337 | 25245752 | 5’-flanking | C/T | 0.066 | 1 | rs12877218,rs12864635,rs17474223,rs11619780,rs34611608,rs34137051,rs17080934, rs1530530,rs11616959,rs12871557,rs11620423,rs11619885,rs3803221,rs17080979, rs9553410,rs3759456,rs12872010,rs12871180,rs17081048,rs12873366,rs12869327,  rs12870584 |
| rs1867767 | 25262014 | intron | A/G | 0.055 | 0.897 | / |
| rs2071490 | 25265139 | coding | C/T | 0.185 | 0.999 | rs2071490,rs7981616,rs7997850,rs7996486,rs7996324,rs7318083,rs2071491, rs2071492, rs735770,rs1867769,rs735772,rs2070812,rs895424,rs895425, rs2070809,rs7988856,rs7986361,rs1867768,rs2863259,rs1867766,rs2070811, rs2070814,rs963985,rs2070813,rs963983,rs17081058,rs11616730,rs9553420, rs9553421,rs2289898,rs3816332,rs9553423,rs9553424,rs9553425,rs17081055, rs9581151,rs7323194 |
| rs12872010 | 25265271 | coding | T/C | 0.071 | 0.994 | rs12871180,rs17081048,rs12873366,rs12869327,rs12870584,rs77066059,rs41305040, rs12860747,rs12868533,rs17080979,rs11619885,rs11620423,rs12871557,rs1530530, rs34137051,rs34611608,rs11619780,rs10507337,rs12877218,rs12864635,rs17474223, rs17080934,rs11616959,rs3803221, rs9553410 |
| rs963984 | 25266932 | coding | A/C | 0.097 | 1 | rs3783060,rs118100366 |
| rs2289909 | 25283596 | coding | T/C | 0.023 | 0.999 | rs2289909,rs79244869,rs76072821,rs117171936,rs74962364,rs79840129, rs75004722,rs117418822, rs2497589,rs1539083 |

SNP ID is a GenBank ID number (NCBI). Position and location type were taken from the most recent human genome sequence assemblies (NCBI Build 37.3). Allele and call frequencies were calculated in the studied population. Tagged SNPs present an r²>0.80 with selected *ATP12A* markers. SNP, single-nucleotide polymorphism; ID, identification number; bp, base-pairs; MAF, Minor Allele Frequency. Bad cluster is referred to poor quality genotyping calls as represented in GenomeStudio software (illumina).

Table S2 Characteristics of Participants by Sex

|  | Clinical measurements | |  |  | Echocardiographic measurements | |
| --- | --- | --- | --- | --- | --- | --- |
| Characteristic | Women (*n=*626) | Men  (*n=*540) |  | Characteristic | Women  (*n=*626) | Men  (*n=*540) |
| *Anthropometrics* |  |  |  | *Conventional echocardiography* |  |  |
| Age (years) | 50.4±14.8 | 48.6±15.7* |  | LV internal diameter, diastole (cm) | 4.82±0.39 | 5.25±0.43† |
| Height (cm) | 162.6±6.8 | 175.3±7.2† |  | Interventricular septum, diastole (cm) | 0.92±0.15 | 1.03±0.16 |
| Weight (kg) | 70.6±14.5 | 82.3±12.3† |  | Posterior wall, diastole (cm) | 0.85±0.13 | 0.95±0.13† |
| Body mass index (kg/m²) | 26.7±5.2 | 26.8±3.8 |  | LV mass index (g/m²) | 84.4±19.3 | 99.5±21.3† |
| Systolic pressure (mmHg) | 128.9±20.7 | 132.0±16.2 |  | Ejection fraction (%) | 64.4±6.33 | 62.1±6.70† |
| Diastolic pressure (mmHg) | 79.5±10.9 | 82.7±10.0† |  | TDI s’ peak (cm/s) | 8.42±1.35 | 9.24±1.52† |
| Heart rate (beats/minute) | 64.2±9.7 | 61.0±10.8† |  |  |  |  |
| *Questionnaire data* |  |  |  | *Diastolic function* |  |  |
| Current smoking, *n* (%) | 122 (19.5) | 123 (22.8) |  | Transmitral E peak (cm/s) | 76.4±15.7 | 70.6±15.7† |
| Drinking alcohol, *n* (%) | 113 (18.1) | 304 (56.3) † |  | Transmitral A peak (cm/s) | 66.5±17.9 | 59.4±16.4† |
| Hypertensive, *n* (%) | 271 (43.3) | 251 (46.5) |  | Transmitral E/A ratio | 1.24±0.46 | 1.29±0.50 |
| Treated for hypertension, *n* (%) | 185 (29.6) | 130 (24.1)* |  | TDI e’ peak (cm/s) | 11.0±3.51 | 11.3±3.65 |
| *Biochemical data* |  |  |  | TDI a’ peak (cm/s) | 9.68±2.04 | 10.1±2.18* |
| Blood glucose (mmol/l) | 4.96±0.92 | 5.18±1.12* |  | e’/a’ ratio | 1.28±0.67 | 1.23±0.69 |
| Serum creatinine (µmol/l) | 73.4±13.1 | 89.9±17.4† |  | E/e’ ratio | 7.47±2.40 | 6.67±1.97† |
| Serum total cholesterol (mmol/l) | 5.37±1.01 | 5.18±1.02* |  | Composite diastolic score | -0.071±2.06 | -0.077±2.09 |

Values are mean (±SD) or number of subjects (%). LV, left ventricle; TDI, Tissue Doppler Imaging. Significance of the sex difference: **P*≤0.05; †*P*≤0.001. Blood pressure was the average of five consecutive auscultatory readings.

**Table S3: Characteristics of participants by rs10507337 genotypes**

| Clinical measurements | | |  | Echocardiographic measurements | | |
| --- | --- | --- | --- | --- | --- | --- |
| Characteristic | TT  (*n*=1017) | TC or CC  (*n*=149) |  | Characteristic | TT  (*n*=1017) | TC or CC  (*n*=149) |
| *Anthropometrics* |  |  |  | *Conventional echocardiography* |  |  |
| Female sex, *n* (%) | 547 (53.8) | 79 (53.0) |  | LV internal diameter, diastole (cm) | 5.02±0.46 | 5.05±0.48 |
| Age (years) | 49.5±15.2 | 49.6±15.5 |  | Interventricular septum, diastole (cm) | 0.97±0.16 | 0.98±0.18 |
| Height (cm) | 168.5±9.4 | 168.1±9.7 |  | Posterior wall, diastole (cm) | 0.90±0.14 | 0.89±0.15 |
| Weight (kg) | 76.1±14.7 | 75.4±15.1 |  | LV mass index (g/m²) | 91.3±21.3 | 92.5±23.8 |
| Body mass index (kg/m²) | 26.8±4.6 | 26.7±5.0 |  | Ejection fraction (%) | 63.4±6.54 | 62.8±7.05 |
| Systolic pressure (mmHg) | 130.2±18.6 | 131.6±20.2 |  | TDI s’ peak (cm/s) | 8.78±1.50 | 8.94±1.45 |
| Diastolic pressure (mmHg) | 81.2±10.7 | 79.7±10.1 |  |  |  |  |
| Heart rate (beats/minute) | 63.0±10.3 | 60.9±10.4* |  | *Diastolic function* |  |  |
| *Questionnaire data* |  |  |  | Transmitral E peak (cm/s) | 73.3±16.0 | 76.4±15.3* |
| Current smoking, *n* (%) | 217 (21.3) | 28 (18.8) |  | Transmitral A peak (cm/s) | 63.4±17.5 | 61.8±17.9 |
| Drinking alcohol, *n* (%) | 372 (36.6) | 45 (30.2) |  | Transmitral E/A ratio | 1.25±0.47 | 1.35±0.50* |
| Hypertensive, *n* (%) | 457 (44.9) | 65 (43.6) |  | TDI e’ peak (cm/s) | 11.1±3.53 | 11.9±3.81* |
| Treated for hypertension, *n* (%) | 277 (27.2) | 38 (25.5) |  | TDI a’ peak (cm/s) | 9.93±2.11 | 9.62±2.18 |
| *Biochemical data* |  |  |  | TDI e’/a’ ratio | 1.24±0.67 | 1.38±0.75* |
| Blood glucose (mmol/l) | 5.05±1.07 | 5.11±0.92 |  | E/e’ ratio | 7.12±2.26 | 6.96±2.15 |
| Serum creatinine (µmol/l) | 81.1±17.7 | 80.2±14.3 |  | Composite diastolic score | -0.058±2.05 | 0.38±2.22* |
| Serum total cholesterol (mmol/l) | 5.29±1.03 | 5.18±0.92 |  |  |  |  |
| Values are mean (±SD) or number of subjects (%). Blood pressure was the average of five consecutive auscultatory readings. LV, left ventricle; TDI, Tissue Doppler Imaging. Significance of the genotype difference: **P*≤0.05 | | | | | | |

Table S4: Adjusted Doppler Diastolic Indexes and Composite Score by *ATP12A* rs10507337 in Subjects without a Previous History of Coronary Heart Disease or Valve Abnormalities

|  |  | rs10507337 | |  | *P* | | |  |
| --- | --- | --- | --- | --- | --- | --- | --- | --- |
| LV diastolic indexes |  | TT  (n=959) | TC or CC  (n=140) |  |  | |  |  |
| Transmitral E peak (cm/s) |  | 71.3±1.80 | 73.2±2.12 |  | | 0.14 | | |
| Transmitral A peak (cm/s) |  | 59.9±1.54 | 58.9±1.79 |  | | 0.32 | | |
| Transmitral E/A ratio |  | 1.29±0.017 | 1.36±0.029 |  | | 0.009 | | |
| TDI e’ peak (cm/s) |  | 10.9±0.25 | 11.6±0.30 |  | | 0.0002 | | |
| TDI a’ peak (cm/s) |  | 9.65±0.24 | 9.53±0.27 |  | | 0.38 | | |
| e’/a’ ratio |  | 1.25±0.027 | 1.35±0.042 |  | | 0.008 | | |
| E/e’ ratio |  | 7.02±0.11 | 6.90±0.18 |  | | 0.41 | | |
| Composite diastolic score |  | 0.060±0.037 | 0.36±0.084 |  | | 0.0005 | | |

Values are least square means ± SE adjusted for family clusters, country, sex, age, body mass index, diastolic blood pressure and heart rate. *P*-values are for the differences between TT homozygotes and C allele carriers. TDI, Tissue Doppler Imaging.

Table S5: Adjusted Doppler Diastolic Indexes and Composite Score by *ATP12A* rs10507337 in Untreated Subjects

|  |  | rs10507337 | |  | *P* | | |  |
| --- | --- | --- | --- | --- | --- | --- | --- | --- |
| LV diastolic indexes |  | TT  (n=740) | TC or CC  (n=111) |  |  | |  |  |
| Transmitral E peak (cm/s) |  | 72.5±2.02 | 74.9±2.36 |  | | 0.081 | | |
| Transmitral A peak (cm/s) |  | 56.9±1.73 | 55.4±1.99 |  | | 0.17 | | |
| Transmitral E/A ratio |  | 1.38±0.024 | 1.45±0.036 |  | | 0.014 | | |
| TDI e’ peak (cm/s) |  | 11.6±0.26 | 12.4±0.32 |  | | 0.0002 | | |
| TDI a’ peak (cm/s) |  | 9.46±0.21 | 9.29±0.25 |  | | 0.26 | | |
| e’/a’ ratio |  | 1.37±0.024 | 1.48±0.044 |  | | 0.011 | | |
| E/e’ ratio |  | 6.57±0.052 | 6.46±0.13 |  | | 0.46 | | |
| Composite diastolic score |  | 0.48±0.040 | 0.83±0.093 |  | | 0.0003 | | |

Values are least square means ± SE adjusted for family clusters, country, sex, age, body mass index, diastolic blood pressure and heart rate. *P*-values are for the differences between TT homozygotes and C allele carriers. TDI, Tissue Doppler Imaging.

Table S6: Adjusted LV Echocardiographic Phenotypes by rs10507337

|  |  | rs10507337 | |  | *P* | | |  |
| --- | --- | --- | --- | --- | --- | --- | --- | --- |
| LV phenotypes |  | TT  (n=1017) | TC or CC  (n=149) |  |  | |  |  |
| LV internal diameter (cm) |  | 5.02±0.019 | 5.01±0.036 |  | | 0.83 | | |
| Interventricular septum (cm) |  | 0.98±0.011 | 0.98±0.015 |  | | 0.59 | | |
| Posterior wall (cm) |  | 0.91±0.004 | 0.90±0.010 |  | | 0.42 | | |
| LV mass index (g/m^2^) |  | 92.1±1.54 | 92.4±2.07 |  | | 0.85 | | |
| Ejection fraction (%) |  | 63.4±0.23 | 62.7±0.56 |  | | 0.27 | | |
| TDI s’ peak (cm/s) |  | 8.44±0.23 | 8.63±0.25 |  | | 0.073 | | |

Values are least square means ± SE adjusted for family clusters, country, sex, age, body mass index, diastolic blood pressure and heart rate. P-values are for the differences between TT homozygotes and C allele carriers. TDI, Tissue Doppler Imaging.

| **Table S7: Adjusted Doppler diastolic indexes and composite score by rs12872010 by country and in all centres combined** | | | | | | | | | |
| --- | --- | --- | --- | --- | --- | --- | --- | --- | --- |
| Country | Genotype | LV diastolic indexes adjusted means ± SE | | | | | | | |
|  |  | E peak (cm/s) | A peak (cm/s) | E/A ratio | TDI e’  (cm/s) | TDI a’  (cm/s) | e’/a’ ratio | E/e’ ratio | Composite diastolic score |
| Belgium (*n*=771) | CC (*n*=648) | 75.3±0.57 | 65.2±0.51 | 1.25±0.013 | 11.3±0.090 | 10.2±0.085 | 1.23±0.018 | 7.14±0.065 | -0.087±0.046 |
|  | T allele carriers (*n*=123) | 76.5±1.26 | 63.0±0.011 | 1.33±0.026 | 12.2±0.18 | 10.0±0.16 | 1.38±0.036 | 6.80±0.15 | 0.33±0.090 |
|  | *P* | 0.35 | 0.052 | 0.004 | 2.6x10^-6^ | 0.26 | 0.0002 | 0.040 | 6.3x10^-6^ |
| Italy (*n*=109) | CC (*n*=97) | 68.0±1.74 | 59.3±1.35 | 1.29±0.030 | 10.3±0.19 | 10.2±0.17 | 1.06±0.036 | 7.12±0.20 | -0.26±0.10 |
|  | T allele carriers (*n*=12) | 74.2±4.18 | 58.7±3.83 | 1.23±0.086 | 10.4±0.52 | 9.93±0.48 | 1.05±0.095 | 7.11±0.54 | -0.18±0.27 |
|  | *P* | 0.14 | 0.88 | 0.55 | 0.91 | 0.57 | 0.94 | 0.99 | 0.79 |
| Poland (*n*=143) | CC (*n*=127) | 74.6±1.27 | 58.4±1.05 | 1.36±0.025 | 11.4±0.20 | 9.14±0.15 | 1.37±0.032 | 7.06±0.18 | 0.43±0.086 |
|  | T allele carriers (*n*=16) | 69.7±3.70 | 58.0±2.96 | 1.30±0.074 | 10.5±0.57 | 9.02±0.43 | 1.21±0.094 | 7.10±0.49 | 0.080±0.25 |
|  | *P* | 0.21 | 0.90 | 0.46 | 0.17 | 0.79 | 0.11 | 0.94 | 0.19 |
| Russia (*n*=136) | CC (*n*=130) | 65.7±1.24 | 59.7±1.03 | 1.20±0.030 | 10.3±0.19 | 9.44±0.16 | 1.24±0.044 | 6.95±0.20 | -0.13±0.11 |
|  | T allele carriers (*n*=6) | 63.0±5.27 | 59.3±4.25 | 1.26±0.13 | 10.4±0.75 | 9.55±0.62 | 1.38±0.18 | 6.47±0.84 | 0.09±0.41 |
|  | *P* | 0.62 | 0.93 | 0.65 | 0.93 | 0.86 | 0.45 | 0.58 | 0.60 |
| All participants (*n*=1159) | CC (*n*=1002) | 71.1±1.74 | 60.7±1.50 | 1.27±0.016 | 10.7±0.26 | 9.65±0.24 | 1.23±0.030 | 7.16±0.11 | -0.039±0.036 |
|  | T allele carriers (*n*=157) | 72.1±2.05 | 59.3±1.75 | 1.33±0.027 | 11.3±0.28 | 9.52±0.27 | 1.33±0.042 | 6.90±0.17 | 0.26±0.081 |
|  | *P* | 0.40 | 0.16 | 0.023 | 8.4*10-5 | 0.33 | 0.003 | 0.083 | 0.0003 |
|  | *P** | / | / | 0.10 | 0.001 | / | 0.008 | / | 0.001 |
|  | *P* for heterogeneity | 0.23 | 0.86 | 0.22 | 0.025 | 0.95 | 0.27 | 0.84 | 0.84 |
| Values are least square means ± SE adjusted for family clusters, country, sex, age, body mass index, diastolic blood pressure and heart rate. TDI indicates Tissue Doppler Imaging, SE, standard error. P-values are for the differences between rs12872010 CC homozygotes and T allele carriers. P*-values are for the differences after Bonferroni adjustment for multiple comparisons. | | | | | | | | | |

| **Table S8: Adjusted Doppler diastolic indexes and composite score by rs9553395 by country and in all centres combined** | | | | | | | | | |
| --- | --- | --- | --- | --- | --- | --- | --- | --- | --- |
| Country | Genotype | LV diastolic indexes adjusted means ± SE | | | | | | | |
|  |  | E peak (cm/s) | A peak (cm/s) | E/A ratio | TDI e’  (cm/s) | TDI a’  (cm/s) | e’/a’ ratio | E/e’ ratio | Composite Diastolic Score |
| Belgium (*n*=772) | TT (*n*=549) | 75.5±0.62 | 65.2±0.55 | 1.25±0.014 | 11.3±0.097 | 10.3±0.089 | 1.23±0.019 | 7.12±0.071 | -0.091±0.050 |
|  | C allele carriers (*n*=223) | 75.4±0.94 | 63.9±0.81 | 1.29±0.020 | 11.7±0.14 | 9.99±0.12 | 1.31±0.028 | 7.00±0.11 | 0.15±0.069 |
|  | *P* | 0.95 | 0.15 | 0.071 | 0.011 | 0.027 | 0.006 | 0.34 | 0.001 |
| Italy (*n*=109) | TT (*n*=67) | 68.2±1.96 | 58.4±1.63 | 1.30±0.037 | 10.4±0.23 | 10.3±0.20 | 1.07±0.042 | 7.02±0.23 | -0.21±0.12 |
|  | C allele carriers (*n*=42) | 69.2±2.31 | 60.7±2.09 | 1.25±0.047 | 10.3±0.28 | 10.0±0.26 | 1.04±0.052 | 7.28±0.29 | -0.32±0.15 |
|  | *P* | 0.70 | 0.40 | 0.41 | 0.68 | 0.45 | 0.67 | 0.48 | 0.57 |
| Poland (*n*=143) | TT (*n*=109) | 74.4±1.35 | 58.2±1.10 | 1.36±0.027 | 11.3±0.21 | 9.13±0.16 | 1.35±0.035 | 7.03±0.19 | 0.43±0.091 |
|  | C allele carriers (*n*=34) | 73.3±2.43 | 59.1±1.92 | 1.32±0.048 | 11.1±0.38 | 9.13±0.28 | 1.34±0.064 | 7.16±0.32 | 0.28±0.17 |
|  | *P* | 0.69 | 0.67 | 0.40 | 0.61 | 0.99 | 0.90 | 0.70 | 0.41 |
| Russia (*n*=137) | TT (*n*=104) | 65.0±1.33 | 59.2±1.08 | 1.19±0.032 | 10.3±0.20 | 9.50±0.17 | 1.24±0.047 | 6.89±0.21 | -0.14±0.11 |
|  | C allele carriers (*n*=33) | 66.7±2.26 | 61.6±1.83 | 1.22±0.056 | 10.3±0.34 | 9.35±0.28 | 1.28±0.079 | 7.00±0.36 | -0.096±0.18 |
|  | *P* | 0.51 | 0.25 | 0.71 | 0.97 | 0.63 | 0.57 | 0.79 | 0.82 |
| All participants (*n*=1161) | TT (*n*=829) | 71.0±1.79 | 60.6±1.49 | 1.27±0.016 | 10.7±0.25 | 9.70±0.24 | 1.22±0.031 | 7.13±0.11 | -0.042±0.039 |
|  | C allele carriers (*n*=332) | 71.4±1.88 | 60.4±1.57 | 1.29±0.020 | 10.9±0.27 | 9.49±0.25 | 1.28±0.035 | 7.10±0.14 | 0.094±0.057 |
|  | *P* | 0.69 | 0.99 | 0.30 | 0.064 | 0.044 | 0.015 | 0.77 | 0.029 |
|  | *P** | / | / | / | / | 0.19 | 0.10 | / | 0.28 |
|  | *P* for heterogeneity | 0.86 | 0.22 | 0.13 | 0.30 | 0.85 | 0.30 | 0.68 | 0.11 |
| Values are least square means ± SE adjusted for family clusters, country, sex, age, body mass index, diastolic blood pressure and heart rate. TDI indicates Tissue Doppler Imaging, SE, standard error. *P*-values are for the differences between rs9553395 TT homozygotes and C allele carriers. *P**-values are for the differences after Bonferroni adjustment for multiple comparisons. | | | | | | | | | |

| **Table S9: Adjusted Doppler diastolic indexes and composite score by rs2071490 by country and in all centres combined** | | | | | | | | | |
| --- | --- | --- | --- | --- | --- | --- | --- | --- | --- |
| Country | Genotype | LV diastolic indexes adjusted means ± SE | | | | | | | |
|  |  | E peak (cm/s) | A peak (cm/s) | E/A ratio | TDI e’ (cm/s) | TDI a’  (cm/s) | e’/a’ ratio | E/e’ ratio | Composite diastolic score |
| Belgium (*n*=776) | TT (*n*=556) | 75.6±0.62 | 65.0±0.56 | 1.26±0.014 | 11.5±0.098 | 10.2±0.091 | 1.26±0.019 | 7.08±0.07 | -0.022±0.050 |
|  | C allele carriers (*n*=220) | 75.0±0.95 | 64.6±0.81 | 1.27±0.020 | 11.3±0.14 | 10.2±0.12 | 1.24±0.028 | 7.10±0.11 | -0.050±0.069 |
|  | *P* | 0.55 | 0.63 | 0.70 | 0.31 | 0.80 | 0.58 | 0.92 | 0.71 |
| Italy (*n*=109) | TT (*n*=59) | 67.8±2.04 | 57.9±1.75 | 1.31±0.039 | 10.2±0.24 | 9.83±0.22 | 1.10±0.045 | 7.28±0.25 | -0.17±0.13 |
|  | C allele carriers (*n*=50) | 69.5±2.15 | 60.8±1.89 | 1.24±0.042 | 10.5±0.25 | 10.6±0.23 | 1.01±0.047 | 6.94±0.27 | -0.35±0.14 |
|  | *P* | 0.52 | 0.28 | 0.19 | 0.28 | 0.017 | 0.17 | 0.33 | 0.34 |
| Poland (*n*=143) | TT (*n*=76) | 74.2±1.62 | 58.4±1.29 | 1.35±0.033 | 11.3±0.25 | 9.01±0.19 | 1.36±0.042 | 7.06±0.22 | 0.41±0.11 |
|  | C allele carriers (*n*=67) | 74.1±1.74 | 58.3±1.38 | 1.36±0.035 | 11.3±0.27 | 9.27±0.20 | 1.34±0.045 | 7.06±0.23 | 0.38±0.12 |
|  | *P* | 0.97 | 0.97 | 0.93 | 1.00 | 0.32 | 0.84 | 0.99 | 0.85 |
| Russia (*n*=137) | TT (*n*=79) | 65.1±1.52 | 60.1±1.26 | 1.19±0.037 | 10.3±0.23 | 9.45±0.19 | 1.26±0.054 | 6.93±0.24 | -0.14±0.13 |
|  | C allele carriers (*n*=58) | 65.8±1.77 | 59.3±1.46 | 1.20±0.043 | 10.3±0.27 | 9.48±0.22 | 1.23±0.062 | 6.89±0.28 | -0.12±0.15 |
|  | *P* | 0.75 | 0.67 | 0.91 | 0.80 | 0.91 | 0.76 | 0.90 | 0.94 |
| All participants (*n*=1165) | TT (*n*=770) | 71.2±1.80 | 60.7±1.51 | 1.28±0.018 | 10.8±0.25 | 9.59±0.24 | 1.24±0.030 | 7.14±0.12 | 0.008±0.041 |
|  | C allele carriers (*n*=395) | 71.0±1.83 | 60.6±1.54 | 1.27±0.019 | 10.7±0.26 | 9.72±0.031 | 1.22±0.03 | 7.10±0.13 | -0.038±0.052 |
|  | *P* | 0.79 | 0.92 | 0.81 | 0.53 | 0.19 | 0.30 | 0.69 | 0.44 |
|  | *P* for heterogeneity | 0.85 | 0.72 | 0.58 | 0.52 | 0.17 | 0.78 | 0.81 | 0.88 |
| Values are least square means ± SE adjusted for family clusters, country, sex, age, body mass index, diastolic blood pressure and heart rate. TDI indicates Tissue Doppler Imaging, SE, standard error. *P*-values are for the differences between rs2071490 TT homozygotes and C allele carriers. | | | | | | | | | |

| **Table S10: Adjusted Doppler diastolic indexes and composite score by rs963984 by country and in all centres combined** | | | | | | | | | |
| --- | --- | --- | --- | --- | --- | --- | --- | --- | --- |
| Country | Genotype | LV diastolic indexes adjusted means ± SE | | | | | | | |
|  |  | E peak (cm/s) | A peak (cm/s) | E/A ratio | TDI e’  (cm/s) | TDI a’  (cm/s) | e’/a’ ratio | E/e’ ratio | Composite diastolic score |
| Belgium (*n*=777) | CC (*n*=655) | 75.5±0.58 | 65.0±0.52 | 1.26±0.013 | 11.4±0.092 | 10.2±0.086 | 1.24±0.018 | 7.10±0.065 | -0.049±0.28 |
|  | A allele carriers (*n*=122) | 75.0±1.27 | 64.4±1.08 | 1.28±0.026 | 11.4±0.18 | 10.1±0.15 | 1.30±0.037 | 7.03±0.15 | 0.052±0.090 |
|  | *P* | 0.71 | 0.57 | 0.38 | 0.74 | 0.30 | 0.16 | 0.64 | 0.28 |
| Italy (*n*=109) | CC (*n*=80) | 67.2±1.83 | 58.9±1.49 | 1.28±0.034 | 10.3±0.21 | 10.0±0.19 | 1.08±0.039 | 7.07±0.22 | -0.23±0.11 |
|  | A allele carriers (*n*=29) | 72.3±2.67 | 60.1±2.51 | 1.29±0.056 | 10.5±0.33 | 10.7±0.31 | 1.00±0.061 | 7.26±0.35 | -0.32±0.18 |
|  | *P* | 0.080 | 0.68 | 0.85 | 0.49 | 0.068 | 0.23 | 0.63 | 0.68 |
| Poland (*n*=143) | CC (*n*=109) | 74.1±1.38 | 59.0±1.12 | 1.34±0.027 | 11.2±0.22 | 9.15±0.16 | 1.34±0.035 | 7.12±0.19 | 0.35±0.094 |
|  | A allele carriers (*n*=34) | 74.0±2.38 | 56.6±1.83 | 1.39±0.048 | 11.4±0.37 | 9.08±0.27 | 1.40±0.062 | 6.87±0.30 | 0.53±0.16 |
|  | *P* | 0.97 | 0.26 | 0.40 | 0.61 | 0.82 | 0.39 | 0.45 | 0.33 |
| Russia (*n*=137) | CC (*n*=105) | 65.7±1.36 | 59.5±1.11 | 1.21±0.033 | 10.3±0.21 | 9.37±0.17 | 1.26±0.048 | 7.00±0.21 | -0.088±0.11 |
|  | A allele carriers (*n*=32) | 64.4±2.41 | 60.5±1.96 | 1.14±0.059 | 10.3±0.36 | 9.77±0.29 | 1.20±0.084 | 6.64±0.38 | -0.28±0.20 |
|  | *P* | 0.64 | 0.67 | 0.059 | 1.00 | 0.23 | 0.50 | 0.41 | 0.39 |
| All participants (*n*=1166) | CC (*n*=949) | 71.1±1.78 | 60.7±1.49 | 1.27±0.016 | 10.8±0.25 | 9.64±0.24 | 1.23±0.029 | 7.15±0.11 | -0.017±0.037 |
|  | A allele carriers (*n*=217) | 71.2±1.94 | 60.6±1.62 | 1.28±0.023 | 10.7±0.28 | 9.67±0.25 | 1.25±0.036 | 7.06±0.15 | 0.015±0.068 |
|  | *P* | 0.96 | 0.90 | 0.71 | 0.97 | 0.81 | 0.53 | 0.50 | 0.66 |
|  | *P* for heterogeneity | 0.38 | 0.64 | 0.24 | 0.82 | 0.11 | 0.29 | 0.77 | 0.47 |
| Values are least square means ± SE adjusted for family clusters, country, sex, age, body mass index, diastolic blood pressure and heart rate. TDI indicates Tissue Doppler Imaging, SE, standard error. P-values are for the differences between rs963984 CC homozygotes and A allele carriers. | | | | | | | | | |
